# Sanatorium Treatment

**Published:** 1920-05-01

**Authors:** 


### Sanatorium Treatment.

THE future administration of sanatorium treatment, which has been removed from the ægis of the National Insurance Act and is to be entrusted to local authorities, was the subject of a statement by the Minister of Health on Tuesday last. He said the reason for the change was that Health Services were contemplated which could more conveniently deal with tuberculosis.

A certain amount of work was already done at the tuberculosis dispensaries provided by the local authorities. The sanatoria had, with few exceptions, been provided by the county councils and county borough councils, and the insurance committees had made arrangements with them for the reception of their cases. It had been found, more and more as a result of the war, that a great deal of the expenditure which had been incurred in sending people to sanatoria was wasted. When a large number of persons went back again to their trade it imposed heavy burdens on their powers, with the result that they relapsed. Thus a very large percentage of people in certain districts who had been to sanatoria had died of the disease later.

Last year they obtained grants from the Treasury which would enable local authorities to provide training as an adjunct to sanatorium treatment, and thus they were able to secure training in alternative occupations. When there was a properly organised training system it had enormously beneficial effects. Where a man could live under decent conditions with an alternative occupation, such as was the case in a colony village, a large number of permanent cures were effected.

They could not deal with this subject unless they had one authority competent to deal with the whole question. Whatever authority they set up—they were working it out now—it would have to be an authority having charge of the different parts of the Health Service. He promised that there should be an opportunity of co-opting on the new authorities members of insurance committees who had special experience of tuberculosis.

